# Gallbladder Cancer Concomitant With Para‐Aortic Paraganglioma: A Case Report

**DOI:** 10.1002/ccr3.71066

**Published:** 2025-09-29

**Authors:** Hisashi Murakami, Satoshi Okubo, Yutaro Naka, Noritaka Kudo, Yutaka Takazawa, Masahiro Kobayashi, Masaru Matsumura, Junichi Shindoh, Masaji Hashimoto

**Affiliations:** ^1^ Department of Gastroenterological Surgery Toranomon Hospital Minato‐Ku Tokyo Japan; ^2^ Department of Pathology Toranomon Hospital Minato‐Ku Tokyo Japan

**Keywords:** differential diagnosis, gallbladder cancer, lymph node metastasis, paraganglioma

## Abstract

Gallbladder cancer often presents a poor prognosis and can cause skip metastasis to the para‐aortic lymph nodes. Accurate diagnosis is essential for appropriate treatment. We present the case of an 86‐year‐old male patient with gallbladder cancer initially suspected to have para‐aortic lymph node metastasis. Intraoperative pathology of a para‐aortic mass revealed that it was a paraganglioma (PGL); both tumors were successfully resected. The patient was discharged on the ninth postoperative day without complications and remained recurrence‐free for 18 months. The final diagnosis was gallbladder adenocarcinoma (pT1a(M)pN0pM0, pStage I) and para‐aortic PGL. Differentiating para‐aortic masses in gallbladder cancer is crucial. Preoperative evaluations, including positron emission tomography‐computed tomography and endoscopic ultrasound‐fine needle biopsy, can enhance diagnostic accuracy. This case underscores the importance of considering PGL as a differential diagnosis of a para‐aortic mass and the value of intraoperative pathology in guiding appropriate surgical interventions.

**Summary**

- Gallbladder cancer can cause skip metastasis to para‐aortic lymph nodes, making accurate diagnosis crucial for treatment.
- When a para‐aortic mass is present with gallbladder cancer, consider paraganglioma and conduct preoperative evaluations.
- Paragangliomas, even without catecholamine symptoms, can be hormone‐secreting and require resection.

## 1 Introduction

Gallbladder cancer has a relatively poor prognosis because its symptoms are difficult to recognize, and it may only be detected after disease progression. Surgery may cure the disease in its early stages; however, the indications for surgery in advanced cancers are controversial. Para‐aortic lymph node metastasis is a poor prognostic factor, and its dissection is not usually indicated in advanced gallbladder cancer [1].

Paraganglioma (PGL) is a non‐epithelial tumor arising from extra‐adrenal paraganglia, 75% of which occur intraperitoneally [2]. Although studies have reported its association with hereditary diseases such as multiple endocrine neoplasia (MEN) type 2A and neurofibromatosis (NF) type 1, PGL concomitant with gastrointestinal cancer has not been reported.

Herein, we report the case of an 86‐year‐old man with gallbladder cancer concomitant with PGL who was thought to have para‐aortic lymph node metastasis preoperatively.

## 2 Case History/Examination

**FIGURE 1:**
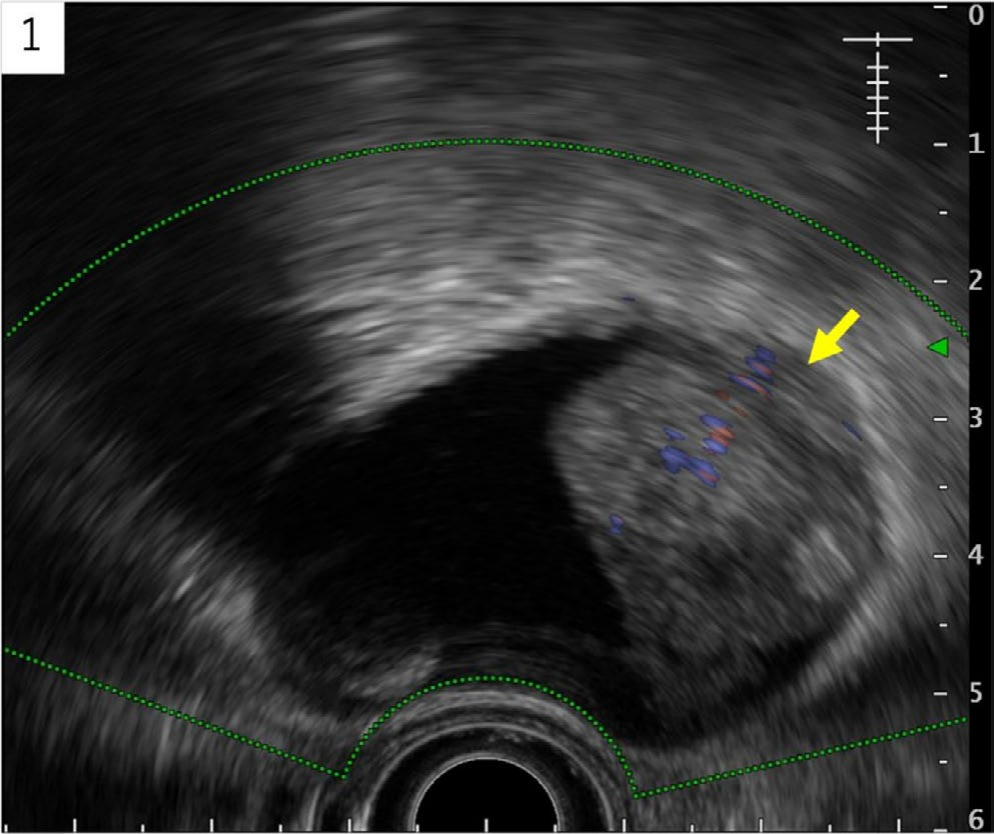
Endoscopic ultrasonography. A 28 mm irregularly‐shaped inhomogeneous, hyperechoic semi‐pedunculated lesion in the funds of gallbladder, and a blood flow signal on the inside by Doppler ultrasonography.

**FIGURE 2:**
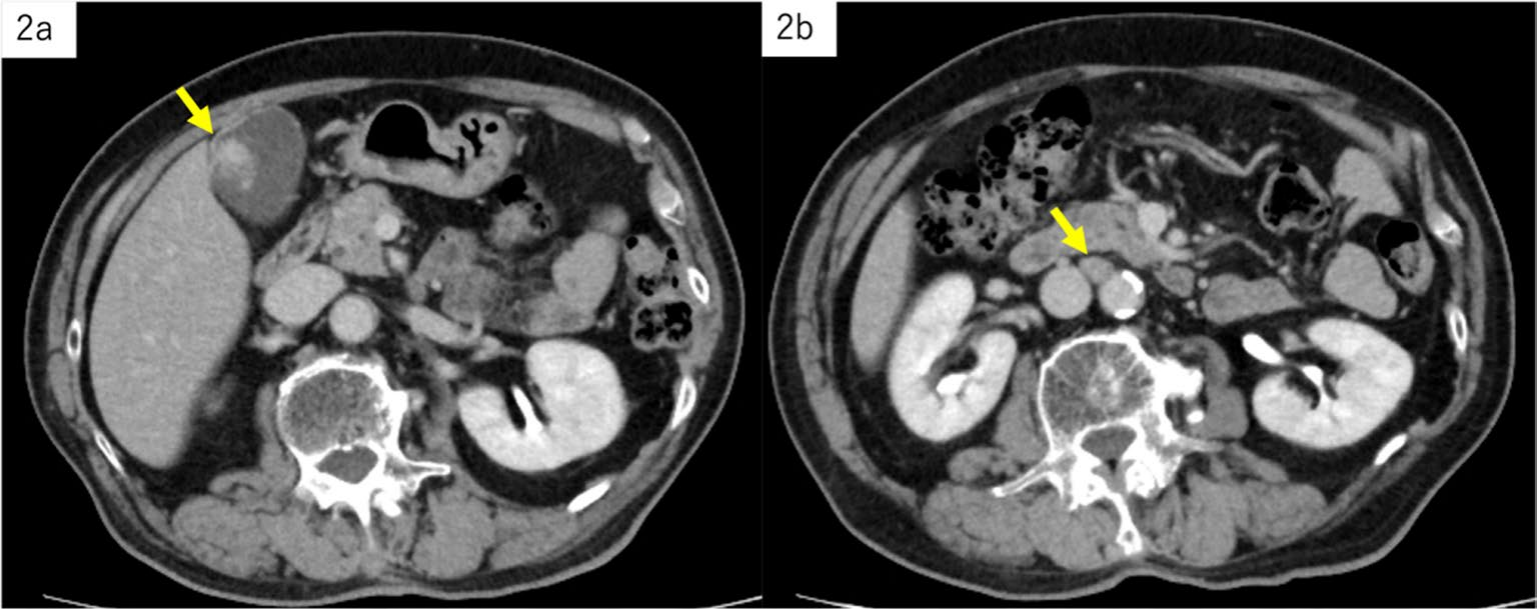
Computed tomography. (a) An inhomogeneous contrast‐affected nodule inside the gallbladder. (b) A 12 mm mass on the right side of the aorta.

**FIGURE 3:**
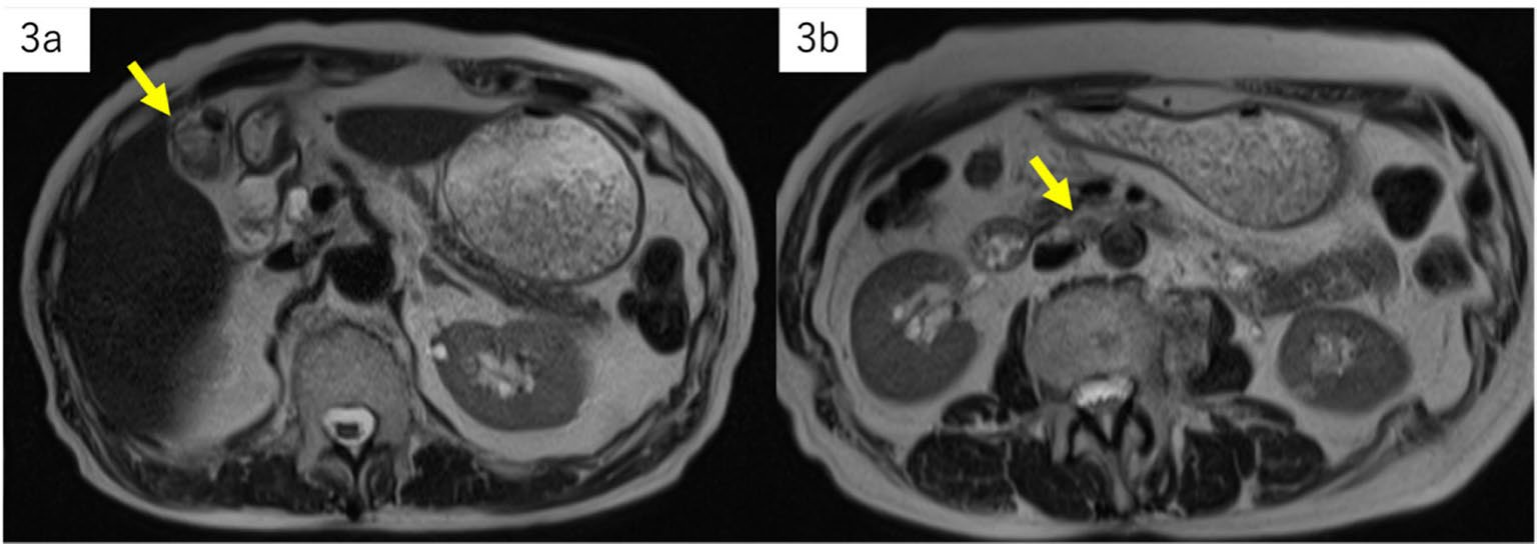
Magnetic resonance imaging. The gallbladder lesion (a) and para‐aortic mass (b) show inhomogeneous signals on T2WI. T2WI, T2 weighted image.

A gallbladder mass was observed in an 86‐year‐old man with a history of alcohol‐associated liver disease during an abdominal ultrasonography follow‐up. Endoscopic ultrasonography showed a 28‐mm‐sized irregularly shaped, inhomogeneous, hyperechoic semi‐pedunculated lesion in the fundus of the gallbladder, and Doppler ultrasonography detected blood flow signal on the inside. The outermost layer was thin and partially obscured; however, there was no evidence of invasion beyond the serosa (Figure 1). Computed tomography (CT) showed an inhomogeneous contrast‐affected nodule inside the gallbladder, and a 12‐mm mass was detected on the right side of the aorta (Figure 2a,b). Magnetic resonance imaging (MRI) showed an inhomogeneous signal on T1‐weighted image (WI) and T2WI and a high signal on diffusion‐weighted imaging in the gallbladder lesion as well as the para‐aortic mass (Figure 3a,b). The tumor depth was expected to be subserous (SS), and gallbladder cancer with para‐aortic lymph node metastasis was suspected based on the imaging findings; however, the lymph nodes within the hepatoduodenal ligament, including the #12c lymph node, were not enlarged.

## 3 Methods

### 3.1 Differential Diagnosis, Investigations, and Treatment

Based on the differential diagnosis of a para‐aortic mass, we considered the possibility of other diseases such as lymphoma and schwannoma, in addition to para‐aortic lymph node metastasis. Our plan was to first perform an intraoperative pathological examination to determine if the para‐aortic mass was a lymph node metastasis, and if not, to proceed with primary resection. Laparoscopic para‐aortic mass resection was performed, and intraoperative pathological examination revealed a neoplastic lesion with irregular nuclei and eosinophilic cytoplasm, but no lymph node metastasis. Distant metastasis of the gallbladder cancer was ruled out, and laparoscopic gallbladder bed resection was performed. An additional intraoperative pathological examination revealed no cancer at the proximal edge of the cystic duct and in the #12c lymph node. Macroscopically, the tumor was a pedunculated lesion, and the tumor depth was expected to be less than the muscularis propria (MP). Furthermore, as the patient was an older adult, a minimally invasive procedure was desired. Therefore, we decided to complete the surgery without additional lymph node dissection and wait for the final pathological diagnosis. The operation time was 191 min, and there was minimal bleeding.

## 4 Conclusions and Results

### 4.1 Outcome and Follow‐Up

**FIGURE 4:**
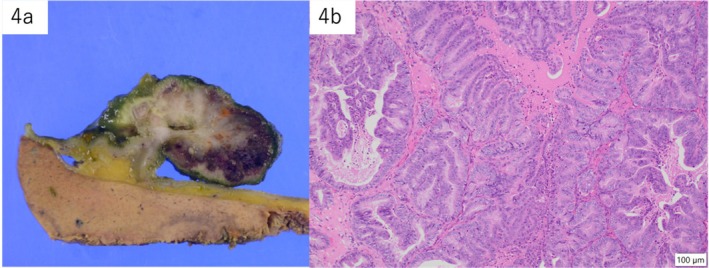
Images of resected gallbladder cancer. (a) Macroscopically, the tumor is a large polypoidal mass located in the fundus. (b) The microscopic examination shows a well‐to‐moderately differentiated tubular adenocarcinoma spreading to the Rokitansky–Aschoff sinus epithelium.

**FIGURE 5:**
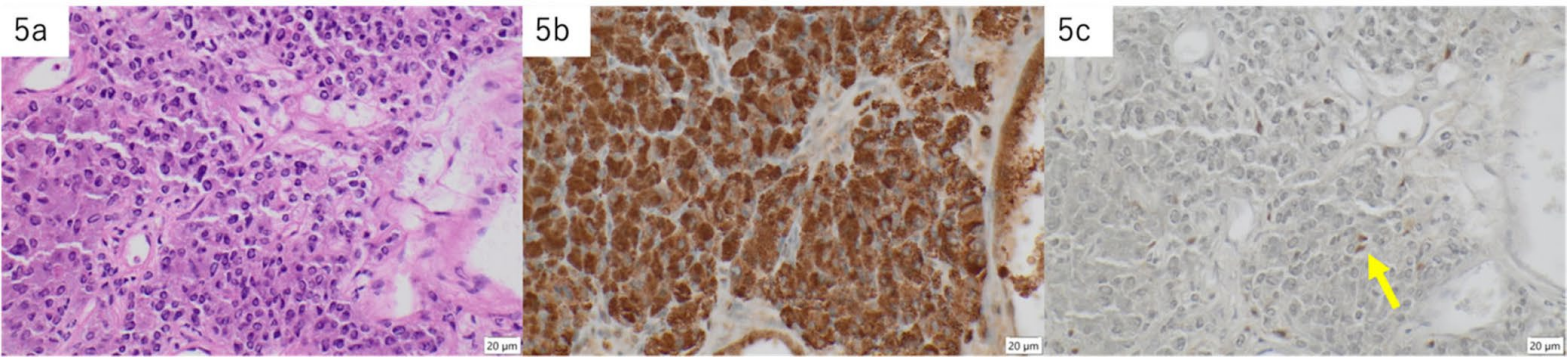
Histological images of the para‐aortic paraganglioma. (a) The tumor cells are epithelioid with abundant granular basophilic cytoplasm (hematoxylin & eosin stain). (b) Immunohistochemically, the tumor cells are positive for chromogranin A (chromogranin A immunostain). (c) S‐100 positive sustentacular cells are observed around the nests (S‐100 immunostain).

Gross examination of the resected gallbladder revealed a 38 × 29 × 20‐mm‐sized polypoid tumor located in the fundus (Figure 4a). A microscopic examination revealed a well‐to‐moderately differentiated tubular adenocarcinoma spreading to the Rokitansky–Aschoff sinus epithelium (Figure 4b). The pathological diagnosis of the gallbladder cancer was adenocarcinoma with pT1a(M)pN0pM0 (pStage I). Microscopically, the resected para‐aortic mass comprised epithelioid cells with abundant granular basophilic cytoplasm arranged in nests (Zellballen pattern). Immunohistochemically, the epithelioid chief cells were positive for chromogranin A, and S‐100‐positive sustentacular cells surrounded the nests. The pathological diagnosis of the para‐aortic mass was a PGL (Figure 5a–c). The patient was discharged on the ninth postoperative day without any apparent complications, including abnormalities in blood pressure or heart rate. The patient was under outpatient observation for 18 months postoperatively; no recurrence was observed.

## 5 Discussion

Gallbladder cancer has a relatively poor prognosis; it is often asymptomatic and detected at an advanced stage. In addition to tumor depth, tumor location, and lymph node metastasis, the number of lymph nodes [3] and the presence of para‐aortic lymph node metastasis [1] are important prognostic factors in gallbladder cancer.

The para‐aortic lymph node is the final point of the lymphatic flow pathway from the gallbladder, drained via the cystic node (#12c lymph node), pericholedochal node, and nodes posterior or superior to the pancreatic head. Approximately 19%–26% of patients with advanced gallbladder cancer have para‐aortic lymph node metastasis [1]. Patients with para‐aortic lymph node metastases are generally recommended to undergo lymph node sampling first, followed by radical resection, as para‐aortic lymph node dissection is likely to provide no survival benefit [1].

The #12c lymph node is considered a sentinel lymph node in gallbladder cancer and serves as a useful indicator of downstream lymph node metastasis. In contrast, lymph flow from the gallbladder also has a direct route from the hilar lymph nodes to the para‐aortic lymph node [4]. Rare cases of skip metastasis to the para‐aortic lymph node have been reported, even in the absence of #12c lymph node metastasis. Birnbaum et al. [5] reported that five such cases (5.7%) of skip metastasis without lymph node metastasis in the hepatoduodenal ligament among 87 cases of patients undergoing D2 dissection. Yasukawa et al. [6] further suggested that the occurrence of skip metastasis may be influenced by tumor location, particularly in tumors located on the liver side of the gallbladder.

Diseases such as malignant lymphomas, schwannomas, and PGLs should be considered in the differential diagnosis of para‐aortic masses, in addition to lymph node metastasis [7]. PGLs are nonepithelial tumors arising from extra‐adrenal paraganglia; 75% of sympathetic PGLs occur intraperitoneally [2], mainly around the aorta, with rare cases reported in the bladder [8] and gallbladder [9]. According to the 5th edition of the World Health Organization Classification of Paragangliomas and Pheochromocytomas, PGLs are classified as non‐epithelial malignancies that can metastasize, with 20%–50% of cases showing metastatic spread [10]. Therefore, surgical resection is the standard treatment, but the possibility of inducing a catecholamine crisis should be carefully monitored, as PGLs may have hormone‐producing potential, even in the absence of preoperative symptoms [11].

There have been no reports of PGLs concomitant with gallbladder cancer, but a case of PGL found as a retroperitoneal tumor 5 years after gastric cancer surgery has been reported [12]. In the case of para‐aortic PGL concomitant with gastrointestinal cancer, it is important to differentiate it from lymph node metastasis and to make a diagnosis preoperatively to avoid misdiagnosis of the stage. The lesion in the present case was thought to be a lymph node metastasis of gallbladder cancer, but as there was no invasion of other organs by gallbladder cancer, and the lymph node mass was solitary with no findings suspicious of other lymph node metastases, we were of the opinion that the para‐aortic mass could be a separate lesion, and R0 resection could be performed if intraoperative pathology ruled out lymph node metastasis. To make a more accurate diagnosis, preoperative positron emission tomography‐CT should have been performed to evaluate distant metastases or preoperative endoscopic ultrasound‐guided fine needle aspiration to evaluate para‐aortic masses. However, in this case, concerns regarding the invasiveness of the procedure, the possibility of inconclusive results, and the potential delay in initiating treatment for gallbladder cancer led to the decision to prioritize surgical intervention. Although PGL was not suspected preoperatively, the patient was discharged safely without catecholamine excess symptoms during the perioperative period. If PGL is suspected, urinary vanillylmandelic acid and 123I‐MIBG scintigraphy are recommended because it is difficult to differentiate PGL from metastases on contrast‐enhanced CT, positron emission tomography‐CT, or MRI.

In conclusion, gallbladder cancer is a disease with a relatively poor prognosis that can cause skip metastasis in the para‐aortic lymph nodes; therefore, accurate diagnosis is crucial for effective treatment. When a para‐aortic mass is observed concomitantly with intra‐abdominal cancer, differential diagnoses should include not only distant metastasis but also paraganglioma. Current guidelines recommend the resection of all paragangliomas. Moreover, clinicians should consider that these tumors can be hormone‐secreting even in the absence of symptoms caused by increased catecholamine levels.

## Author Contributions

**Hisashi Murakami:** conceptualization, writing – original draft. **Satoshi Okubo:** writing – review and editing. **Yutaro Naka:** writing – review and editing. **Yutaka Takazawa:** writing – review and editing. **Noritaka Kudo:** writing – review and editing. **Masahiro Kobayashi:** writing – review and editing. **Masaru Matsumura:** writing – review and editing. **Junichi Shindoh:** writing – review and editing. **Masaji Hashimoto:** writing – review and editing.

## Ethics Statement

All methods were performed in accordance with relevant guidelines and regulations, including the Declaration of Helsinki.

## Consent

Formal written informed consent was obtained from the patient for the publication of this report, in compliance with the journal's patient consent policy.

## Conflicts of Interest

The authors declare no conflicts of interest.

## Data Availability

The data that support the findings of this study are available upon request from the corresponding author.
